# Enhancing User Experience in Virtual Reality Through Optical Flow Simplification with the Help of Physiological Measurements: Pilot Study

**DOI:** 10.3390/s26020610

**Published:** 2026-01-16

**Authors:** Abdualrhman Abdalhadi, Nitin Koundal, Mahdiyeh Sadat Moosavi, Ruding Lou, Mohd Zuki bin Yusoff, Frédéric Merienne, Naufal M. Saad

**Affiliations:** 1 Centre for Intelligent Signal and Imaging Research (CISIR), Department of Electrical and Electronics Engineering, Universiti Teknologi PETRONAS (UTP), Seri Iskandar 32610, Malaysia; nitin_22005110@utp.edu.my (N.K.); mzuki_yusoff@utp.edu.my (M.Z.b.Y.); naufal_saad@utp.edu.my (N.M.S.); 2 LISPEN, Arts et Métiers Institute of Technology, 71100 Chalon Sur Saone, France; mahdiyehsadat.moosavi@ensam.eu (M.S.M.); ruding.lou@ensam.eu (R.L.); frederic.merienne@ensam.eu (F.M.)

**Keywords:** optical flow, cybersickness, virtual reality, electroencephalography

## Abstract

The use of virtual reality (VR) has made significant advancements, and now it is widely used across a range of applications. However, consumers’ capacity to fully enjoy VR experiences continues to be limited by a chronic problem known as cybersickness (CS). This study explores the feasibility of mitigating CS through geometric scene simplification combined with electroencephalography (EEG)-based monitoring. According to the sensory conflict theory, this issue is caused by the discrepancy between the visually induced self-motion (VIMS) through immersive displays and the real motion the vestibular system detects. While prior mitigation strategies have largely relied on hardware modifications or visual field restrictions, this paper introduces a novel framework that integrates geometric scene simplification with EEG-based neurophysiological activity to reduce VIMS during VR immersion. The proposed framework combines EEG neurophysiology, allowing us to monitor users’ brainwave activity and cognitive states during virtual immersion experience. The empirical evidence from our investigation shows a correlation between CS manifestation and neural activation in the parietal and temporal lobes. As an experiment with 15 subjects, statistical differences were significantly different with P= 0.001 and large effect size η2=0.28, while preliminary trends suggest lower neural activation during simplified scenes. Notably, a decrease in neural activation corresponding to reduced optic flow (OF) suggests that VR environment simplification may help attenuate CS symptoms, providing preliminary support for the proposed strategy.

## 1. Introduction

Virtual reality (VR) technology has witnessed rapid advancements, leading to its widespread adoption in various domains such as gaming, training, and medical applications [1]. Despite its potential, one of the significant challenges holding back the full utilization of VR is cybersickness (CS) [2]. CS is a phenomenon that shares similarities with motion sickness [3]. Interestingly, CS can be triggered solely by visual stimuli in the absence of corresponding physical movement. The heightened visual stimulus in such environments provides users with a greater wealth of environmental information, making it more challenging to ignore the conflicting sensory cues. Previous study has explored various factors contributing to CS, including visual vestibular conflicts, display latency, and user susceptibility [4].

In contrast to prior work that has mainly relied on hardware adjustments [5], visual field restrictions [6,7], or post-processing [8], the novelty of our study lies in combining geometric simplification of virtual scenes with electroencephalography (EEG)-driven analysis of optical flow (OF). This integration enables both objective detection of CS markers and dynamic adjustment of visual complexity to mitigate symptoms. To contextualize this contribution, it is important to note that CS is commonly studied through user evaluations, since it is inherently a subjective experience. Some methods are intrusive, like using sensors to track body signals [9], while others rely on what participants say during or after the session, which may not always be accurate [10]. Our study suggested an alternative way to distinguish CS is by analyzing visual features like OF, which tracks motion between video frames to understand visual movement. A study by Smith [11] introduced a metric combining OF and entropy to assess the potential of VR environments to induce CS. The findings indicated that environments with higher OF entropy were more likely to cause CS symptoms. This suggests that increased complexity and intensity of visual motion contribute to discomfort during VR interaction. The limitation of his work is that OF data were extracted from a stationary viewpoint, thus capturing only the environmental motion while excluding user-induced head movements that typically contribute to CS. Although widely researched in VR, OF has some limitations. It includes horizontal, vertical, and magnitude components, which all affect the viewer differently but happen at the same time, making it hard to study separately [12,13]. Also, the amount of the OF can vary depending on how fast the user moves or how the environment is designed, and a high number of the OF events do not always mean the experience will cause CS [11]. To address the limitations of relying solely on OF for predicting CS, a pilot study proposes a new approach to objectively characterize self-motion experiences in head-mounted display (HMD) virtual environments (VE). The method aims to identify CS inducing features as part of an iterative design process, allowing for the evaluation and refinement of virtual scenes before user testing. It combines OF metrics with an analysis of visual motion in both central and peripheral fields of view (FOV). This approach also incorporates scene simplification in the peripheral view and compares simple versus complex environments, alongside the use of physiological signals to enhance prediction accuracy. One of the well-known neurophysiological measurements is EEG, which can be used to investigate the neural correlations of CS, offering insights into the brain’s response during VR exposure [14,15]. For instance, changes in EEG power spectral densities in specific frequency bands have been associated with the onset of CS symptoms [16,17]. Several approaches have been proposed to mitigate CS, such as adjusting FOV restrictions, incorporating rest frames, and employing motion prediction algorithms [18,19]. While there are practices that can alleviate CS within VE, such as narrowing the FOV [7] or incorporating background images [20], these methods tend to reduce the sense of presence and immersion, thereby continuing to hinder the widespread adoption of VR. To address these challenges, it becomes imperative to employ effective strategies for the enhancement of visual perception in VR to mitigate the CS.

CS detection can be achieved by analyzing physiological signals collected via sensors such as Electrocardiogram and Galvanic Skin Response (GSR) [21]. Neurophysiological devices employing GSR, EEG, and Photoplethysmography techniques are used to obtain accurate, non-invasive insights within psychiatry. Real-time measurements and reduction in CS with the help of EEG measurement have also shown promising outcomes [22]. However, there remains a need for more effective and objective methods to detect and alleviate CS [23,24].

Subjective self-reports can be used to assess the presence of virtual reality induced symptoms and effects (VRISE). The Simulator Sickness Questionnaire (SSQ) [25], which has become the standard tool to quantify VRISE in VR, is the most often-used subjective questionnaire for assessing VR-induced symptoms and effects. SSQ has been utilized in various works [26,27]. However, not many studies have introduced physiological measurement methods, for instance, fNIRS or EEG.

EEG is widely used in brain–machine interface (BMI) applications due to its affordability, high temporal resolution, and compatibility with diverse devices. EEG provides significant advantages over other brainwave measurement techniques, such as electrocorticography (ECoG) and magnetoencephalography (MEG). Primarily, EEG is non-invasive, eliminating the need for electrode insertion into the brain [28]. A study by Krokos [15] recorded EEG during a Vection inducing VR fly-through and compared CS against the baseline. They reported significant increases in delta (1–4 Hz), theta (4–7 Hz), and alpha (7–13 Hz) power correlating with joystick-reported sickness. This supports EEG as a continuous, real-time marker, in contrast to self-reported questionnaire data. A study by Hasan [29] emphasizes the critical importance of assessing CS through tools like the SSQ and evaluating presence metrics to gauge user discomfort and engagement levels in VR environments. Recent work by Demirel [30] proposed a real-time continuous estimation method combining dry electrode EEG + head motion, using multitaper spectral analysis and Long Short-Term Memory (LSTM) algorithm, which tracks dynamic CS levels without calibration, overcoming limitations of post VR questionnaires like SSQ and enabling more granular tracking. These works demonstrate the potential of EEG for dynamic monitoring, but they stop short of coupling detection with adaptive VR design. By combining EEG analysis with geometric simplification and OF adjustments, the present study addresses this gap, offering both objective detection and proactive mitigation within immersive VR environments.

In VR, discrepancies between visual motion cues and physical movement can disrupt perception, leading to discomfort. FOV plays a significant role in this context. These OF patterns have the potential to induce pronounced Vection [31], which contradicts the motion cues perceived by the vestibular system. Reducing FOV can mitigate motion sickness by limiting peripheral motion cues, yet it may also diminish immersion [6]. To address this, techniques like dynamic FOV adjustment have been proposed, which adaptively narrow the FOV during rapid movements to balance comfort and immersion [32]. Another strategy involves implementing rest frames stable visual references within the VR environment, such as a virtual nose or cockpit that help anchor the user’s perception and reduce sensory conflict [33]. While this strategy can alleviate CS, this often does so at the expense of immersion by restricting the user’s view or adding artificial anchors. The proposed approach differs by aiming to reduce CS without compromising immersion, through geometric simplification of scene complexity guided by EEG based monitoring. Innovative approaches like peripheral teleportation create a stable peripheral view aligned with the user’s physical motion, further mitigating CS while preserving immersion [34]. These findings underscore the importance of carefully designing visual cues and reference frames in VR to enhance user comfort and experience. A study conducted by [35] employed geometric simplification to diminish OF, aiming to mitigate CS in the context of VR. The same author also proposed the use of geometric deformation, which would reduce the OF, causing less CS in the VR environment [36], The study did not incorporate EEG, eye-tracking, or physiological indicators, e.g., heart rate, skin conductance, which could have provided deeper insight into the sensory conflict and its neural processing. In contrast, our work advances this line of research by combining scene simplification with EEG-driven analysis, enabling both detection and mitigation of cybersickness. Another study by [18] suggested that mitigating CS in VR could be achieved through the implementation of Adaptive FOV Limitations, which restrict the OF experienced by users. Consequently, the phenomenon of CS in VR can be attributed to the OF resulting from locomotion, particularly when users engage in virtual movement without corresponding physical motion. In the context of wide FOV displays, geometric mismatches in FOV restriction may lead to suboptimal occlusion or undesirable visual artifacts. On the other hand, the proposed method avoids such artifacts by directly simplifying the geometry of the virtual environment, allowing a more natural reduction in the OF that aligns with user perception. Given the exploratory nature of this work and the limited number of participants, the present study should be regarded as a pilot investigation aimed at validating the feasibility of integrating EEG feedback with geometric simplification to mitigate cybersickness.

## 2. Materials and Methods

To investigate the neurophysiological correlations of CS induced by varying levels of OF, three geometrically distinct VE were developed using Unity 3D. These environments included (1) a high OF scenario featuring complex visual details, (2) a moderately simplified scene with a 50% reduction in geometric complexity, and (3) a fully simplified scene with a 100% geometric reduction.

Upon wearing the HMD, participants were immersed in a maze-like VE while seated in a virtual automated vehicle resembling a coaster on a flat track. Their movement involved passive navigation through the VE, which featured straight segments and lateral turns without vertical undulations. This automated locomotion was consistent across all three visual complexity conditions. EEG data were recorded throughout the exposure to analyze the relationship between OF intensity and neural responses. The experimental design enabled the evaluation of how increasing visual complexity and OF levels influenced CS symptoms and corresponding EEG patterns.

A visual vigilance task was intentionally included to maintain a consistent level of attention and ensure continuous visual fixation. Participants were required to count randomly positioned paintings on the walls and report the total post-trial; this protocol was implemented to prevent the eye-closure behaviors observed in previous studies. Importantly, the task did not involve manual controllers or motor interaction; only the geometric complexity and corresponding OF were manipulated. This controlled design ensured that any observed differences in EEG activation or cybersickness trends could be attributed primarily to variations in visual motion complexity rather than to motor execution or physical workload.

### 2.1. Participants

This study engaged 15 healthy male participants, aged from 22 to 40 years old [29.40 ± 4.85]. While the sample size is limited, this study aimed at exploring conceptual feasibility and analyzing initial EEG and CS-related data in VR [37,38,39]. All the participants are free from ophthalmologic conditions, following the acquisition of their written informed consent. Prior to the experiment, participants abstained from consuming substances such as alcohol, drugs known to induce nausea or headaches, and caffeine. To ensure the integrity of EEG data, participants were instructed to minimize head movements and remain silent during the EEG recording sessions, which were challenged by some technical difficulties associated with EEG data acquisition. This study was approved by the Ethics Committee of the University Pendidikan Sultan Idris (No. UPSI/PPPI/PYK/ETIKA(M)/Jld. 19(89)).

### 2.2. Apparatus

**Figure 1:**
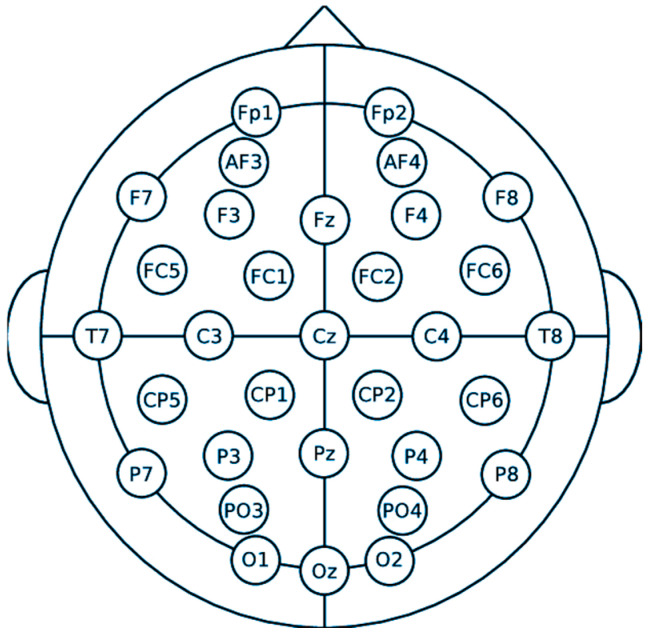
International 10–20 system for 32 channels connection [41].

EEG signals were recorded using the eego™ sports EE-201 amplifier (AntNeuro, eemagine Medical Imaging Solutions GmbH, Gubener Str., Berlin, Germany). Electrode placement followed the international 10–20 system [40], with electrodes positioned as shown in Figure 1. A forehead was placed at the Ground (GND) electrode, while the reference electrode was located at CPz. All electrode impedances were carefully maintained below 10 kΩ to ensure signal quality. During the experiment, participants wore an Oculus Quest 2 VR headset.

Using EEG, brain activity will be recorded during each scenario. Pre-processing steps will remove artifacts, followed by feature extraction using methods such as Independent Component Analysis (ICA), complex Morlet wavelet transforms, and power spectral density (PSD) analysis to identify time-frequency patterns associated with CS.

### 2.3. Pre-Processing

EEG data were pre-processed using the open-source EEGLAB toolbox v2025. 1.0 in MATLAB R2024a, with a sampling rate of 512 Hz. To attenuate slow drifts and high-frequency noise, a band-pass finite impulse response (FIR) filter was applied with cutoff frequencies of 1–40 Hz, using a Hamming windowed sinc filter. Power line noise at 50 Hz was removed using the Clean Line plugin, applied to ICA components using a sliding window (4 s length, 1 s step) and a spectral smoothing factor of 100. Noisy or corrupted EEG channels were visually identified and interpolated using spherical spline interpolation, only when clearly necessary. The data were then re-referenced to the average of all channels to minimize spatial noise. Finally, Independent Component Analysis (ICA) was performed to identify and remove artefactual components related to eye movements and muscle activity. All pre-processing procedures were consistently applied across all participants to ensure data quality and comparability.

### 2.4. Geometrical Simplification and Optical Flow Analysis

**Figure 2:**
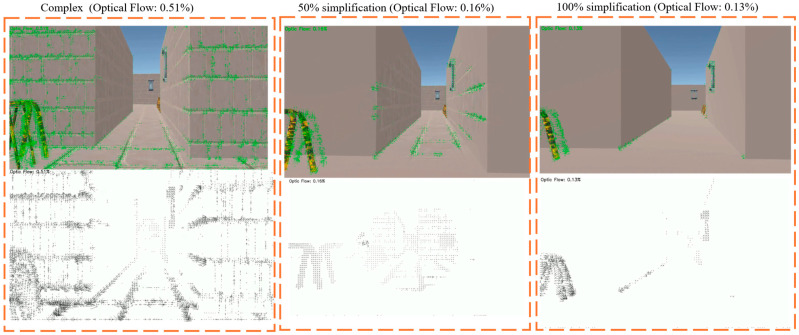
VR scene showing optical flow simulation: (**Left column**): complex scene with the highest optical flow, (**Middle column**): simplified scene with 50% reduction, and (**Right column**): fully simplified scene with the least optical flow (100% simplification).

To investigate the impact of geometrical complexity on user discomfort in VR, three distinct scene conditions were developed as shown in Figure 2. Each scene varied in terms of geometry complexity and texture detail, thereby influencing the amount of OF perceived during navigation Figure 2 (second raw).

The OF measured using Gunnar Farnebäck algorithm [42]. Farnebäck approximates the neighborhood of each pixel in an image as a quadratic polynomial:

(1) $$f(x)\approx x^{T}Ax+b^{T}x+c$$

where x = (x,y) pixel position, A is a symmetric matrix second-order derivative, b is a vector first-order derivative, and c is a scalar offset. The coefficients are estimated from a weighted least squares fit to the signal values in the neighborhood.

Suppose between two frames I_1 and I_2, a small neighborhood is translated by a flow vector. Then, the polynomial coefficients change accordingly:

(2) $$f_{2}\;(x)=f_{1}\;(x-d)$$

So, we compare polynomial expansions of $f_{1}$ and $f_{2}$, and the goal is to find the displacement d that best aligns the neighborhoods (minimizing error between polynomials). The optimal displacement vector d is obtained by minimizing the difference in local polynomial representations between frames:

- A1, b1, c1 from frame 1.
- A2, b2, c2 from frame 2.

We then solve

(3) $$d=\arg min\int_{x\in \Omega }{\left[f_{1}\left(x\right)-f_{2}\left(x+d\right)\right]}^{2}dx$$

Farnebäck’s algorithm applies this displacement estimation densely across the image using image pyramids (multi-scale), smoothing and filtering (Gaussian), and iterative refinement. This gives a dense flow field

(4) flow(x,y)=(u(x,y),v(x,y))

where u and v are the horizontal and vertical displacements, respectively, for each pixel.

The direct application of the Euclidean norm (L2 norm) is used to measure the magnitude of a vector as follows:

(5) $$Magnitude=\sqrt{(dx^{2}+dy^{2})\;}$$

The weighted arithmetic means a standard statistical measure:

(6) $$Weighted\;Avg=\frac{\sum (mag\cdot weight)}{\sum \left(weight\right)}$$

Normalization and scale value to a relative percentage:

(7) $$Percent=\frac{avg_{mag}}{max_{flow}}\times 100$$

The OF is computed using Farneback’s dense OF algorithm from OpenCV using the following python library called cv2.calcOpticalFlowFarneback. The Complex condition, representing a fully detailed scene without any geometric simplification (Figure 2) Left column, exhibited the highest OF at 0.51%. This scenario simulates a visually rich and dynamic environment, which is commonly linked to elevated levels of motion sickness due to increased visual complexity and motion cues. In contrast, the repetitive wall designs significantly reduced to 0.16% and 0.13%, respectively. This simplification aims to minimize the perceptual load and reduce sensory conflict. In Figure 2 middle and right column, 50% and 100% Simplified conditions eliminate architectural textures and introduce uniform ones. These variations in OF directly correlate with user discomfort, as summarized in Table 1. The Complex scene recorded the highest OF scores (mean: 2.13, median: 2.16). The Simplified conditions show a substantial decline in OF. For instance, the Simplified 50% scene report average OF scores of 0.69. The Simplified 100% condition further decreases OF (mean: 0.51) along with flow percentage (0.13%). Reducing visual motion through geometric simplification and the removal of dynamic stimuli is shown to significantly alleviate symptoms of CS, making it a valuable design consideration for enhancing immersive VR experiences [35,43].

### 2.5. Data Collection Protocols

**Figure 3:**
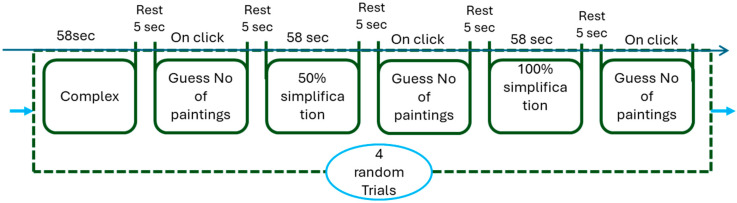
Data collection timeline and the number of trials.

After confirming the validity and reliability of the experimental protocol and obtaining ethical consent, EEG was integrated with VR technology. The experimental procedure commenced with a baseline recording of 120 s, followed by the first task (complex scene) of 58 s duration, a subsequent rest period of 10 s, a second task (50% simplified scene) lasting 58 s and same for the last task (100% simplified scene), and a final rest interval of 10 s. This sequence was repeated across 2 trials, culminating in total duration of approximately 13.8 min. The relatively short exposure duration (58 s per scene) was deliberately chosen to minimize participant discomfort and prevent severe cybersickness during this pilot study. Longer exposure times will be implemented in future experiments to better capture the gradual onset of cybersickness symptoms. Figure 3 illustrates the timeline for all the scenes. Prior to the commencement of EEG recording, participants underwent training to familiarize themselves with the VR environment, including trial runs. Participants were instructed to sit comfortably and minimize blinking and movement as much as possible during the EEG recording sessions to ensure the accuracy and reliability of the data collected. All procedural conditions, alongside potential effects, as well as the participants’ autonomy to withdraw at any moment, were thoroughly communicated. The entire experimental session, inclusive of EEG electrode placement, was conducted within a time frame of less than one hour.

## 3. Results and Discussion

The following findings represent preliminary observations based on a moderate participant cohort.

### 3.1. Brain Topography

**Figure 4:**
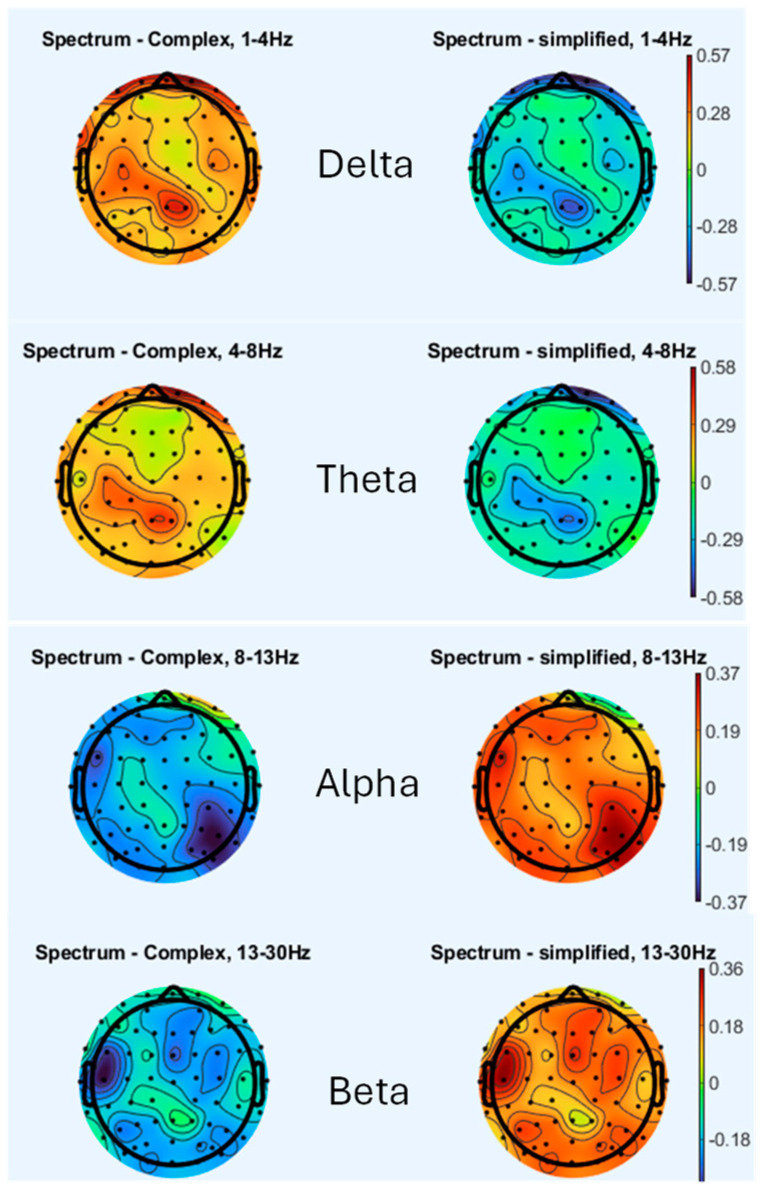
Brain topography results in the complex, 100% simplification.

Figure 4 presents the EEG spectral power topography across four frequency bands (delta, theta, alpha, and beta) for two experimental conditions: condition 1 (100% simplification) and condition 2 (complex scene). When analyzing these EEG bands, a notable observation is that lower amplitude values, which are closer to baseline levels, are typically associated with fewer symptoms of CS. This trend appears most pronounced in condition 1, where reduced delta and theta power density indicates a reduction in the participant’s susceptibility to CS. This reduction in neural activity suggests that less visually stimulating conditions, such as those in condition 1, evoke fewer symptoms related to motion sickness.

Conversely, condition 3, which represents a more complex scene, seems to be associated with greater amplitude fluctuations across multiple regions, particularly in the Delta, Theta, and Beta bands. These fluctuations suggest a stronger neural response to more complex visual stimuli, which correlate with increased symptoms of CS. The greater variability in power density reflects an overstimulation of the sensory system, which is often linked to discomfort and disorientation in VE. Regarding the alpha wave band, higher levels of alpha power have been widely associated with relaxation and reduced sensory input processing. The elevated alpha power in certain conditions, such as those seen in condition 1, indicates a more relaxed or comfortable state for participants. This observation aligns with established research showing that increases in alpha power are linked to states of relaxation and comfort [44,45,46,47].

### 3.2. Statistical Analysis and Results

**Figure 5:**
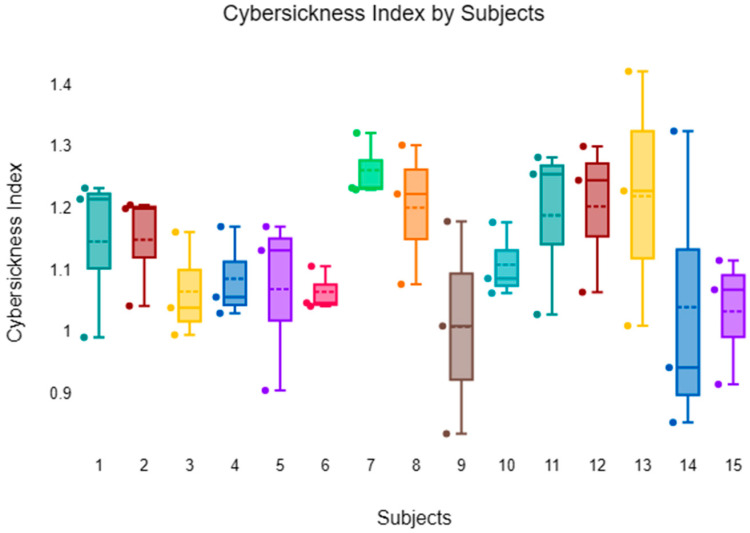
EEG wave-based condition and averaged PSD for fifteen subjects.

Figure 5 visualizes the CS ratings across 15 subjects (S1 to S15) under various conditions, including different levels of scene simplification and environmental factors. Preliminary observations indicate no significant differences between the subjects; however, there are significant differences between conditions, suggesting that all participants experienced similar effects during the experiment. Across subjects, the complex scene appeared to evoke stronger CS symptoms than the Simplified conditions, with the 100% simplification condition consistently showing the lowest CS ratings, whereas these patterns align with prior EEG studies [15,48].

The key focus of this analysis lies in the role of alpha, delta, theta, and beta waves in relation to CS, as the Gamma band remains unchanged across all scenes and has thus been excluded from our CS index. This omission is consistent with prior research, where the gamma band has similarly been found to exhibit minimal variation during conditions associated with CS [14]. A significant observation is the behavior of the alpha band, particularly in the more complex scenes. Alpha power shows marked suppression during the “complex” condition (0.81) in Table 2. This pattern of alpha suppression during vection or high sensory conflict is consistent with prior findings [47]. In this context, the suppression of alpha power is indicative of heightened neural engagement, possibly explaining the increased CS reported in more complex VE. This elevation in alpha power during rest is consistent with the association of alpha waves with relaxation and reduced sensory input processing, further supporting the idea that higher alpha power corresponds to a more comfortable and relaxed state for participants.

Four tests (Kolmogorov–Smirnov, Smirnov with Lilliefors correction [49], Shapiro–Wilk [50], and Anderson–Darling [51]) produced *p*-values greater than 0.05, as shown in Table 3, indicating that there is no significant deviation from normality. Therefore, it is appropriate to proceed with statistical methods that assume the normality of the data based on these test results.

A one-way ANOVA was conducted to examine the effect of scene condition (Complex, 50%, and 100% simplifications) on the Cybersickness Index. The analysis revealed a statistically significant difference between the groups, F (2, 42) = 8.03, *p* = 0.001. The effect size, calculated as eta squared (η2), was 0.28, indicating that approximately 28% of the variance in cybersickness scores can be explained by the level of scene simplification; this represents a large effect size. Descriptive statistics indicated that cybersickness scores were highest in the Complex condition (M = 1.19, SD = 0.12), followed by the Simp50 condition (M = 1.14, SD = 0.11), and lowest in the Simp100 condition (M = 1.03, SD = 0.12).

Post hoc comparisons using the Bonferroni correction indicated that the Complex condition (M = 1.19, SD = 0.12) elicited significantly higher cybersickness scores than the 100% simplification condition (M = 1.03, SD = 0.12), t = 3.91, *p* = 0.001. Similarly, the 50% simplification condition (M = 1.14, SD = 0.11) showed significantly higher scores than the 100% condition, t = 2.72, *p* = 0.028. However, no statistically significant difference was found between the Complex and 50% conditions (*p* = 0.73).

**Figure 6:**
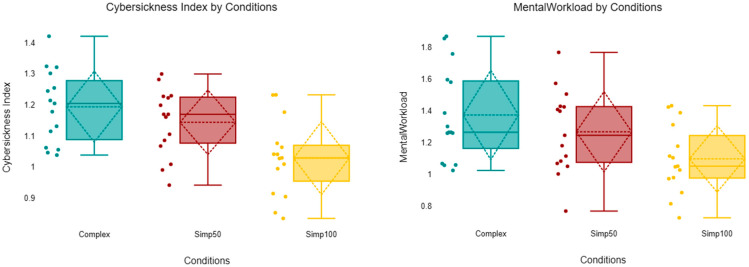
Averaged power density for the CS and Mental Workload Index.

Table 4 and Figure 6 present average data across all trials, providing insight into the levels of CS, mental workload, across different experimental conditions. The box plots reveal a clear downward trend in cybersickness severity as the level of scene simplification increases. The Complex condition exhibited the highest central tendency (Median = 1.20, Mean = 1.19) and the widest spread of scores, indicating that this condition consistently induced higher levels of cybersickness among participants. The 50% simplification condition showed a slightly reduced central tendency (Median = 1.17, Mean = 1.14) but maintained a distribution range that heavily overlaps with the Complex condition, visually corroborating the non-significant difference found in the post hoc analysis (*p* = 0.73). In contrast, the 100% simplification condition demonstrated a marked reduction in cybersickness, with the lowest median (1.03) and mean (1.03) scores. The interquartile range or 100% is notably lower than that of the Complex condition, reinforcing the significant main effect found in the ANOVA and the significant post hoc difference between these two groups (*p* = 0.001).

When analyzing the mental workload and CS, it becomes evident that participants experienced the highest levels of mental workload in the “Complex” condition (mean = 1.37) and 50% Simplification (mean = 1.27) as shown in Figure 6, which corresponds to the elevated CS scores.

### 3.3. Simulator Sickness Questionnaire

**Figure 7:**
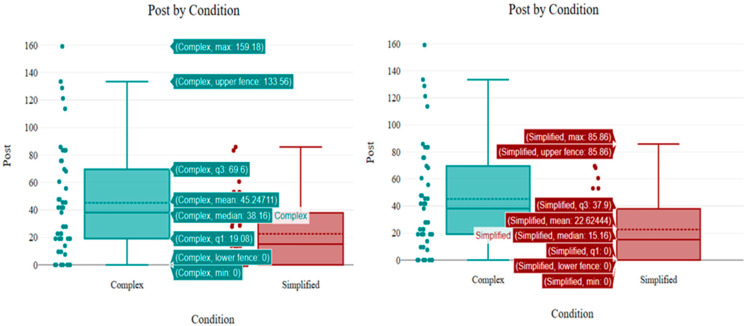
Distribution of post-experiment SSQ scores for the Complex and Simplified conditions.

Figure 7 presents the distribution of post-experiment SSQ scores for the Complex and Simplified conditions. The box plots demonstrate a substantial reduction in reported sickness symptoms in the Simplified condition compared to the Complex condition. Participants in the Complex condition reported considerably higher sickness levels, with a median score of 38.16 and a mean of 45.25. The wide interquartile range (IQR: 19.08 to 69.60) and the high upper fence (133.56) indicate significant variability, with some participants experiencing severe symptoms, with maximum score of 159.18.

In contrast, the Simplified condition resulted in markedly lower sickness scores, showing a median of 15.16 and a mean of 22.62—approximately half the average severity of the Complex group.

The distribution is also much tighter (IQR: 0 to 37.90), suggesting that participants in the simplified scene consistently felt better. Notably, the lower quartile and minimum scores for the Simplified group are 0, indicating that at least 25% of participants in this group experienced negligible to no symptoms.

### 3.4. Limitations

While this study presents a promising approach to mitigating CS through geometric simplification and EEG-based measures, several limitations must be acknowledged. The most significant constraint is the limited sample size, with 15 participants included in the study. This small cohort reduces the statistical power of the findings and limits the generalizability of the results. 

The experimental conditions themselves were also relatively moderate (each scene lasting only 58 s), which may not fully reflect the effects of prolonged VR exposure that often intensifies CS. Furthermore, the study focused mainly on geometric simplification, but other contributing factors such as texture and color dynamics were not fully isolated or controlled for. 

### 3.5. Future Works

Increasing the sample size to enhance statistical robustness and enable more definitive conclusions about condition specific effects. Extending the exposure duration to better simulate realistic VR usage and observe long term impacts on CS and brain activity. Also incorporating multimodal physiological sensors, e.g., GSR, PPG alongside EEG to provide a more comprehensive picture of user discomfort and cognitive load. Exploring machine learning models to predict CS in real time based on EEG features, allowing for adaptive VR content delivery.

## 4. Conclusions

In conclusion, this study explored discomfort mitigation in VR environments through a framework that integrates EEG measures with geometric simplification. This approach allowed monitoring and analysis of user’s cognitive states during different VR conditions. Preliminary observations indicate a relationship between CS and increased neural activity, particularly in the parietal and temporal lobes, and suggest that geometric simplification may help reduce neural activation associated with CS. Trends also indicate a correlation between higher OF in the scene and elevated CS levels. Being conclusive due to the small sample size, they provide exploratory evidence supporting the potential of combining EEG-based monitoring with OF simplification as a novel approach to CS mitigation. This study contributes new insights into the design of VR experiences that aim to balance immersion and user comfort. For future work, expanding the sample size and diversity will be essential to validating the preliminary findings. Moreover, investigating adaptive real-time geometric simplification strategies based on individual user responses could lead to more personalized and effective VR discomfort reduction.

## Figures and Tables

**Table 1:** Optical flow means and median average.

Condition	Mean	Median
Complex	2.13	2.16
simplified 50%	0.69	0.58
simplified 100%	0.51	0.38

**Table 2:** EEG wave-based condition and averaged PSD for all subjects.

Scenes	Delta	Theta	Alpha	Beta	Gamma	Total
Complex	1.02	0.99	0.81	0.92	0.87	0.92
Simp50	0.98	0.98	0.86	0.93	0.92	0.93
Simp100	0.99	0.97	0.94	1	0.98	0.98

**Table 3:** Tests of normality across scene conditions: Kolmogorov–Smirnov, Shapiro–Wilk, and Anderson–Darling results for each level of geometric simplification.

	Statistics	*p*
Kolmogorov–Smirnov	0.08	0.887
Kolmogorov–Smirnov (Lilliefors Corr.)	0.08	0.58
Shapiro–Wilk	0.98	0.728
Anderson–Darling	0.34	0.483

**Table 4:** CS, Mental Workload Index (MWI), and tress score for six conditions.

Conditions	MWI	CS	Std
Complex	1.37	1.19	0.12
50% Simplification	1.27	1.14	0.11
100% Simplification	1.10	1.03	0.12

## Data Availability

Data supporting reported results can be provided upon request.

## Abbreviations

The following abbreviations are used in this manuscript:

VR	Virtual Reality
EEG	Electroencephalography
FNIRS	Function Near-Infrared Spectroscopy
CS	Cybersickness
FOV	Field Of View
OF	Optical Flow
VRISE	Virtual Reality induced symptoms and effects
SSQ	Simulator Sickness Questionnaire
HMD	Head-mounted display
BMI	Brain–machine interface
